# Interactions between the nitrogen-fixing cyanobacterium *Trichodesmium* and siderophore-producing cyanobacterium *Synechococcus* under iron limitation

**DOI:** 10.1093/ismeco/ycae072

**Published:** 2024-05-25

**Authors:** Xumei Sun, Yan Xiao, Chengwen Yong, Hansheng Sun, Shuangqing Li, Hailong Huang, Haibo Jiang

**Affiliations:** School of Marine Sciences, Ningbo University, 818 Fenghua Road, Ningbo, Zhejiang, 315211, People’s Republic of China; Southern Marine Science and Engineering Guangdong Laboratory (Zhuhai), 1 Jintang Road, Zhuhai, Guangdong, 519000, People’s Republic of China; School of Life Sciences, Central China Normal University, 152 Luoyu Road, Wuhan, Hubei, 430079, People’s Republic of China; School of Marine Sciences, Ningbo University, 818 Fenghua Road, Ningbo, Zhejiang, 315211, People’s Republic of China; School of Marine Sciences, Ningbo University, 818 Fenghua Road, Ningbo, Zhejiang, 315211, People’s Republic of China; School of Marine Sciences, Ningbo University, 818 Fenghua Road, Ningbo, Zhejiang, 315211, People’s Republic of China; School of Marine Sciences, Ningbo University, 818 Fenghua Road, Ningbo, Zhejiang, 315211, People’s Republic of China; Southern Marine Science and Engineering Guangdong Laboratory (Zhuhai), 1 Jintang Road, Zhuhai, Guangdong, 519000, People’s Republic of China; School of Marine Sciences, Ningbo University, 818 Fenghua Road, Ningbo, Zhejiang, 315211, People’s Republic of China; Southern Marine Science and Engineering Guangdong Laboratory (Zhuhai), 1 Jintang Road, Zhuhai, Guangdong, 519000, People’s Republic of China; School of Life Sciences, Central China Normal University, 152 Luoyu Road, Wuhan, Hubei, 430079, People’s Republic of China

**Keywords:** Trichodesmium, Synechococcus, Marinobacter, iron deficiency, siderophore

## Abstract

As diazotrophic cyanobacteria of tremendous biomass, *Trichodesmium* continuously provide a nitrogen source for carbon-fixing cyanobacteria and drive the generation of primary productivity in marine environments. However, ocean iron deficiencies limit growth and metabolism of *Trichodesmium*. Recent studies have shown the co-occurrence of *Trichodesmium* and siderophore-producing *Synechococcus* in iron-deficient oceans, but whether siderophores secreted by *Synechococcus* can be used by *Trichodesmium* to adapt to iron deficiency is not clear. We constructed a mutant *Synechococcus* strain unable to produce siderophores to explore this issue. *Synechococcus* filtrates with or without siderophores were added into a *Trichodesmium* microbial consortium consisting of Trichodesmium erythraeum IMS 101 as the dominant microbe with chronic iron deficiency. By analyzing the physiological phenotype, metagenome, and metatranscriptome, we investigated the interactions between the nitrogen-fixing cyanobacterium *Tricodesmium* and siderophore-producing cyanobacterium *Synechococcus* under conditions of iron deficiency. The results indicated that siderophores secreted by *Synechococcus* are likely to chelate with free iron in the culture medium of the *Trichodesmium* consortium, reducing the concentration of bioavailable iron and posing greater challenges to the absorption of iron by *Trichodesmium*. These findings revealed the characteristics of iron-competitive utilization between diazotrophic cyanobacteria and siderophore-producing cyanobacteria, as well as potential interactions, and provide a scientific basis for understanding the regulatory effects of nutrient limitation on marine primary productivity.

## Introduction

Cyanobacteria are the oldest oxygen-releasing photosynthetic microorganisms on Earth and are at the foundation of all material and energy in the marine food chain [1, 2]. Cyanobacteria are dominated by *Prochlorococcus* and *Synechococcus* genera and are widespread in the ocean, contributing up to 50% of primary productivity in oligotrophic open ocean [3–6]. Some cyanobacteria not only perform photosynthetic carbon fixation, but also biological nitrogen (N) fixation, thereby reducing inert atmospheric N_2_ to ammoniacal nitrogen, which participate in the metabolism of countless organisms and provide a major N source in the oceans [6–10].

*Trichodesmium* organisms appear as typical diazotrophic cyanobacteria, with widespread occurrence in vast oligotrophic subtropical and tropical oceans, where they supply a considerable percentage of the bioavailable N input to the global ocean and are particularly important in oligotrophic ocean ecosystems [11–14]. Cyanobacterial growth and metabolism are limited by various physical factors, such as temperature and light, and other than these two factors are mainly limited by the bioavailability of N, phosphorus (P), and iron (Fe) [15–17]. Cyanobacteria may cooperate or compete with each other under nutrient limitation, but we know little about the interactions between different cyanobacteria [18–20].

The capability of diazotrophic cyanobacteria to fix atmospheric N_2_ overcomes the scarcity of other inorganic N sources and is usually limited by the concentration of bioavailable P and Fe in the oligotrophic ocean [21–26]. *Trichodesmium* has employed various strategies to meet Fe and P demands over the long period of its evolution [27–30]. These strategies include modification of its metabolic and physiological characteristics, such as expressing phosphatases to cleave phosphate groups from organic P, using phosphite as a sole phosphorus source for growth, restructuring the proteome broadly, and reducing cell size to adapt to P limitation [22, 29, 31]. Under Fe-deficient conditions, *Trichodesmium* cyanobacteria can regulate cellular Fe quotas, take up different Fe molecules, modulate genes related to Fe uptake, reduce trichome length and growth rates, and increase their surface area to become spherical, thus capturing Fe contained in dust to ameliorate Fe deficiency [27, 32–36].

*Trichodesmium* exists in the ocean as single filaments and macroscopic colonies formed by aggregating filaments (trichomes) [37]. The colonies are usually symbiotic with heterotrophic epibionts and other microorganisms, including diatoms, proteobacteria, and other cyanobacteria [38–40]. Nitrogen, vitamins, trace elements, and other metabolites are exchanged between symbionts and *Trichodesmium*, thus forming diverse interactions to optimize the growth of the entire microbial consortium and contribute to success in oligotrophic systems [41–45]. For example, alkaline phosphatases and acylhomoserine lactones can be produced by *Trichodesmium* consortia members and ultimately assist *Trichodesmium* with P acquisition under P-limited conditions [46, 47]. *Trichodesmium* symbionts also assist in Fe uptake*.* According to recent reports, 23 bacterial strains symbiotic with *Trichodesmium* can produce siderophores in culture to enhance Fe mineral dissolution and bioavailability by assisting in Fe utilization from dust [48, 49]. However, genomic analyses indicate that *Trichodesmium* does not possess known pathways for siderophore synthesis but does have TonB-ExbB-ExbD transporter components that share homology with *Synechocystis* sp. PCC 6803 and provide the possibility of Fe uptake chelated by siderophores secreted by symbionts [18, 50, 51]. Moreover, some symbionts of *Trichodesmium* can utilize more diverse Fe sources than *Trichodesmium*, and some of these symbionts produce siderophores with different chemical properties. It has been reported that these siderophores mainly include hydroxamates, phenolatescatecholates, carboxylates, and others, and they differ in structure, hydrophobicity, and Fe-binding affinity [52]. *Trichodesmium* may differ in terms of acquisition strategies of siderophores with different chemistries [48, 53]. These findings collectively suggest more sophisticated interactions in *Trichodesmium* consortia than we were previously aware of, which provide mutual advantages for the entire consortium and deserve to be explored more deeply.

In the present study, based on metagenomic sequencing, various *Synechococcus* species that can produce synechobactin (a siderophore with Fe-binding functionalities and two α-hydroxamate groups), were found to be symbionts of *Trichodesmium* that were originally isolated from the North Atlantic Ocean and cultured under Fe-deficient conditions in the laboratory. The interaction of *Synechococcus* with *Trichodesmium* under Fe deficiency, whether assisting each other or competing with each other and whether siderophores secreted by *Synechococcus* can be utilized by *Trichodesmium*, is not yet clear. Therefore, to investigate this issue in detail, a *Synechococcus* sp. PCC 7002 mutant lackingsiderophore biosynthesis capability was constructed to explore the interactions under Fe-deficient conditions between *Synechococcus*, which may or may not secrete siderophores, and *Trichodesmium*..

It has been well reported that the siderophore that *Synechococcus* sp. PCC 7002 produces is an amphiphilic synechobactin with a chemical structure that is already clear, consisting of two α-hydroxamate groups, one of which contains a fully saturated fatty acid tail in order of decreasing hydrophobicity [52]. The length of the fatty acid tail varies, with the most common C12, C10, and C8 varieties being named synechobactin A, B, and C, respectively [54]. Previous studies have reported that a gene cluster of *G0025* to *G0018* is responsible for encoding a series of enzymes that catalyze the biosynthesis of siderophores [55]. In this study we constructed and used a mutant by knocking out *G0025* (*sidA*), *G0024* (*sidB*), and *G0023* (*sidC*). Except for the interaction between *Trichodesmium* and *Synechococcus*, the role of another major symbiont, *Marinobacter*, in the adaptation of *Trichodesmium* to Fe deficiency was also analyzed. We found that *Trichodesmium* Fe deficiency was aggravated after treatment with *Synechococcus* filtrate. Metagenomic and metatranscriptomic analyses have demonstrated that the *Synechococcus* filtrate containing siderophores is likely to inhibit the growth of *Trichodesmium* but promotes the growth of *Marinobacter*. Our results revealed a new pattern of Fe competitive utilization between *Synechoccocus* and *Trichodesmium* in *Trichodesmium* consortia.

## Materials and methods

### Cyanobacteria culture and general methods

A *Trichodesmium* consortium originating from the North Atlantic Ocean was obtained from the laboratory of David A. Hutchins at the University of Southern California. This consortium consists of Trichodesmium erythraeum IMS 101 as the dominant microbe as well as *Synechoccocus*, *Marinobacter*, and some other microbes. Stationary culture of the *Trichodesmium* consortium was conducted in YBC-II medium with 250 nmol/L of Fe (Fe replete) or 5 nmol/L of Fe (Fe depleted) and semicontinuously grown at 28°C and 50 μE·m^−2^·s^−1^ (12-hour:12-hour light–dark cycle) in polycarbonate flasks. The axenic *Synechococcus* sp. strain PCC 7002 was obtained from the laboratory of Jindong Zhao at Peking University and was cultured at 110 rpm, at 30°C and 40 μE·m^−2^·s^−1^ (continuous white light) in A^+^ medium (NaCl, 3.0 × 10^−1^ mol/L; KCl, 8.0 × 10^−3^ mol/L; MgSO_4_·7H_2_O, 2.0 × 10^−2^ mol/L; CaCl_2_·2H_2_O, 2.5 × 10^−3^ mol/L; EDTA·2Na, 8.0 × 10^−5^ mol/L; KH_2_PO_4_, 3.7 × 10^−4^ mol/L; V_B12_, 7.4 × 10^−9^ mol/L; FeCl_3_·6H_2_O, 5.0 × 10^−9^ mol/L). Trace-metal clean techniques were applied for the preparation of culture media and both culturing and experimental manipulations.

### Mutant construction

The DNA of the wild-type strain *Synechococcus* sp. PCC 7002 was used as the polymerase chain reaction (PCR) template, and the G0023–25-up (5′-CCGGGATCCGACAAGACAAAGCGCTGG-3′ and 5′-ACGCAGCTGCATAATGACACCCTTTTGAGC-3′) fragment with BamHI and PvuII restriction sites and the G0023–25-down (5′-CGGGGTACCTAGTTTAGAGACTCAAATTCAG-3′ and 5′-TAGTCTAGAGGTCAGTCGGCGACCACA-3′) fragment with KpnI and XbaI restriction sites were amplified for G0023-25-up-1/G0023-25-up-2 and G0023-25-dn-1/G0023-25-dn-2, respectively. The G0023-25-up fragment and G0023-25-down fragment were cloned into the vector pUC19-MCS-C.K2 with restriction enzymes and ligase and kept the same direction of upstream and downstream gene sequences. The recombinant plasmid G0023-25::C.K2 was then transformed into the wild-type strain *Synechococcus* sp. PCC 7002 to produce the siderophore biosynthesis mutant strain Mut-G0023-25, in which G0025 (SidA), G0024 (SidB), and G0023 (SidC) are inactivated [56]. Complete segregation of the mutants was obtained under kanamycin resistance pressure and confirmed by PCR using upstream and downstream primers. The completely segregated mutant was cultured with standard A^+^ medium without kanamycin for investigation of species interactions.

### Fe-deficient *Trichodesmium* treated with the filtrates secreted from *Synechococcus* wild type and Mut-G0023-25 mutant

The *Trichodesmium* consortium was semicontinuously cultured under Fe-depleted conditions (5 nmol/L Fe) for more than 2 months. The Mut-G0023-25 knockout mutant and wild-type *Synechococcus* sp. PCC 7002 were respectively cultured under Fe-depleted conditions (5 nmol/L Fe) both without kanamycin for 1 week, and their filtrates were collected by filtering the culture twice through a 0.22- μm filter membrane. Then the collected filtrates were added into the *Trichodesmium* culture, which had been chronically grown in Fe-depleted medium for more than 2 months. Meanwhile, a blank control was set by adding the same solution of Fe-depleted A^+^ medium (5 nmol/L Fe) into Fe-depleted *Trichodesmium* culture. To determine whether the dominant species of *Trichodesmium* consortium changes, metagenomic sequencing was conducted after Fe-depleted *Trichodesmium* being treated with A^+^ medium and *Synechococcus* filtrate for 5 days.

### Trace-metal clean techniques

All materials used in the Fe-limited cultivation, including conical bottles, PC bottles, and tips, were soaked in 6 mol/L HCl at least 12 hours and rinsed six times with Milli-Q water (18.2 MΩ/cm) to remove residual metal ions. All relevant solutions were using analytical pure reagents without Fe contamination, and all solutions were passed through the chelating column of Chelex-100 to remove the residual Fe before use. Filtering instead of autoclaving was used to avoid Fe contamination in the process of sterilization.

### Chlorophyll *a* content determination

Cultured algae were gathered by centrifugation at 12 000 rpm for 5 min, the precipitate was collected, and 1 mL of 100% methanol was added to the precipitate and gently mixed. The solution was then kept out of light and incubated at 4°C overnight to settle. Centrifugation at 12 000 rpm for 5 min after blending was followed by supernatant absorbance value measurement at 665 nm. The chlorophyll *a* content (μg/mL) was equal to 12 times OD_665_/OD_730_.

### Algal cell content measurement

100 μL of cultured *Trichodesmium* were added to the plankton counting frame (XBC-100CSL) and rested for 1–2 min until all the algal cells settled. A cell was first found at low magnification, then the view was converted to high magnification and the corresponding scale was used (positive differential interference microscope BX53) (Olympus, Tokyo, Japan). The length of the whole algal filament and single cell was determined using measuring software, and the cell number in the counting box was the length of whole algal filament or the length of a single cell.

### Low-temperature fluorescence emission spectrum determination at 77 K

Approximately 400 μL of exponential phase cells were added to glass tubes then pre-cooled and dark-adapted at 4°C, followed by liquid nitrogen flash-freezing until being measured. Cell fluorescence emission spectra were measured using an F-4500 fluorescence spectrophotometer (Hitachi, Tokyo, Japan). According to a previously described study, the excitation wavelength was set to 430 nm and spectra were normalized at an intensity of 720 nm [51]. Results were normalized to cell number.

### RNA extraction and real-time quantitative PCR

Total RNAs of exponential phase cells were extracted as described previously [57]. First-strand cDNA was synthesized by reverse transcription with a HiScript II Q RT SuperMix for qPCR kit (Vazyme, Nanjing, China). Real-time quantitative PCR was conducted with gene-specific primers (*isiA*, 5′-CTGCTCGTTGTTGGTTGACA-3′ and 5′-TCTGCTACGCCATTCAAAGC-3′; *rnpb*, 5′-TGGTAACAGGCATCCCAGATAGATA-3′ and 5′-CGGGTTCTGTTCTCTCAACTCAA-3′). The RT-PCR reaction mixture and process were conducted following instructions in the SYBR Green PCR Master Mix manual (Vazyme, Nanjing, China). Gene expression levels were normalized to *rnpb*. The 2^−ΔΔCt^ method was used to calculate the relative level of mRNA expression [57].

### Metagenomic sequencing

Total DNA was extracted using a FastPure® Bacteria DNA Mini kit (Vazyme, Nanjing, China) following the manufacturer’s protocols. Then the metagenomic DNA was using an Illumina NovaSeq PE150. Duplicate reads, adapter reads, and reads of length <75 bp were first removed. Then the reads were assembled into contigs using MEGAHIT and the assembly data were aligned with sequences (E value, ≤1e^−10^) in the NCBI (National Center for Biotechnology Information) non-redundant protein database [58].

### Metatranscriptome sequencing

The Trizol method was used to extract total RNAs and the Epicentre Ribo-Zero rRNA Removal Kit was used to remove ribosomal RNA [57]. The library was constructed using a NEBNext® Ultra II™ Directional RNA Library Prep Kit for Illumina followed by paired-end sequencing using Illumina HiSeq. After filtering out low-quality reads with fastp and removing ribosome RNAs by alignment with the Rfam database, Hisat2 was used to align the filtered reads to the reference genome and obtain sam/bam files for each sample [59]. RSEM, edgeR, clusterProfiler, Rockhopper were used to calculate the read count for each sample, analyze differential gene expressions, identify functional and pathway enrichment in differential genes, analyze operon and transcription start sites, respectively [60–62].

### Statistical tests

Values were presented as the mean (SD). For 2 group comparison, *p* values were derived from 2-tailed Student t tests or Paired Wilcoxon test to determine statistical significance. For all comparisons, *p* < 0.05 was considered statistically significant difference, while *p* < 0.01 was considered highly significant difference. All experiments were biologically repeated three times.

## Results

### A stable *Trichodesmium* Fe-deficient culture

**Figure 1:**
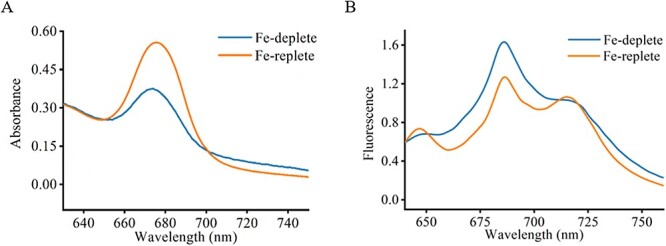
*Trichodesmium* phenotype under Fe-deficient cultivation. (A) *Trichodesmium* absorption spectra at room temperature. The *Trichodesmium* chlorophyll *a* fluorescence absorption peak when cultured under Fe-depleted conditions shifted from 680 to 675 nm compared to that under Fe-replete conditions. (B) 77 K low-temperature fluorescence emission spectroscopy (excitation wavelength at 430 nm) shows the induction level of IsiA.

*Trichodesmium erythraeum* IMS 101 was originally isolated from the North Atlantic Ocean and semi-continuously cultured with YBC-II medium under Fe-depleted condition (5 nmol/L Fe) for more than 2 months (about 15 cycles) in our laboratory (see Methods and materials section). *Trichodesmium erythraeum* IMS 101 formed single filament (trichome) morphology under laboratory conditions (Figure S1A), which has been reported to be symbiotic with microorganisms such as *Synechococcus* [18]. Physiological phenotype measurements showed that the *Trichodesmium* biomass grew in Fe-depleted YBC-II medium, but significantly slower than those grown in Fe-replete medium (250 nmol/L Fe) (Figure S1B). Furthermore, the *Trichodesmium* room temperature chlorophyll *a* fluorescence absorption peak of those grown in Fe-depleted medium shifted from 680 to 675 nm compared with those grown in Fe-replete medium (Fig. 1A), indicating the *Trichodesmium* showed an Fe-deficient phenotype. The 77 K low-temperature fluorescence emission spectroscopy assay shows the characteristic photosystem II complex peak (685 nm and 695 nm) and a photosystem I complex peak (715 nm). The major 77 K emission peak at 685 nm indicates the production of *IsiA*, which is typically characteristic of Fe deficiency and homologous to PsbC (the CP43 of PS II). *Trichodesmium* grown in Fe-depleted medium show an increase in the relative peak at 685 nm and a decrease in the relative peak at 715 nm compared with *Trichodesmium* grown in Fe-replete medium, thus revealing that *isiA* expression is higher under Fe-depleted conditions (Fig. 1B). These data collectively suggest that *Trichodesmium* consortia adapt to a Fe-deficient physiological phenotype after long periods of Fe-depleted cultivation.

### *Synechococcus* and *Marinobacter* are the primary *Trichodesmium* symbionts

**Figure 2:**
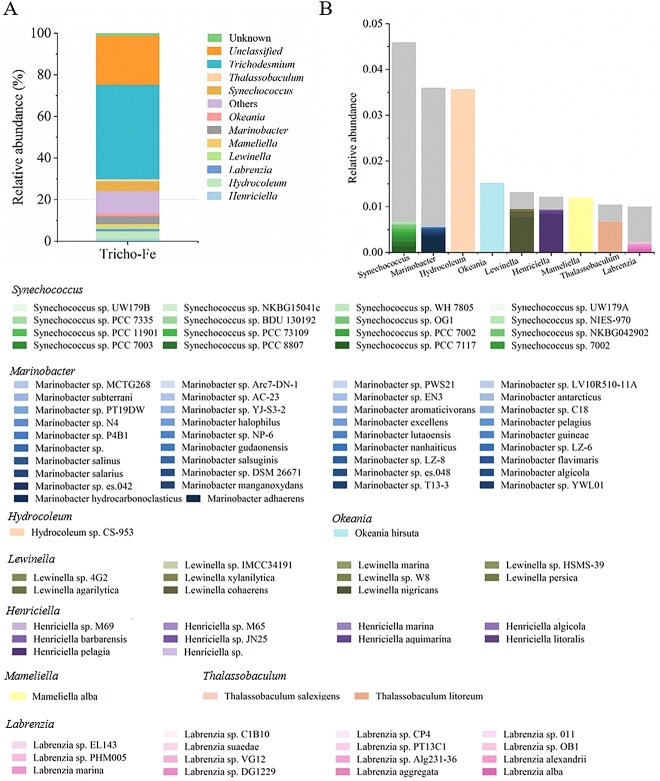
*Trichodesmium* consortium microbial composition under Fe deficiency in the laboratory. (A) Relative abundance of microorganisms in a *Trichodesmium* consortium under Fe-deficient culture. “Others” represent microbial genera with less than 1% of reads. (B) Predominant symbiotic microbial composition at the species taxonomic level. the gray boxes indicate unclassified or unknown microorganisms.

As *Trichodesmium* is a microorganism that originated from the wild, there were diverse microbe symbiosis between *Trichodesmium* and its consortium. To explore the microbial community composition of the *Trichodesmium* consortium that grew in Fe-depleted YBC-II medium for long periods in our laboratory condition, metagenomic sequencing was conducted. Results show *Trichodesmium* to be the dominant genus with about 45.3% relative abundance in the *Trichodesmium* consortium (Fig. 2A). Two other genera of high abundance are *Synechococcus*, which is a genus of photosynthetic autotrophic carbon-fixing cyanobacteria, and the heterotrophic bacteria *Marinobacter*, with the relative abundance of 4.6% and 3.6%, respectively. Relative abundance was 4.6% and 3.6%, respectively (Fig. 2A). At a species taxonomic level, only one species of *Trichodesmium* existed in the consortium, while most *Synechococcus* and *Marinobacter* species were unclassified and unknown in our analysis. Apart from these unknown species, there were 17 *Synechococcus* species and 38 *Marinobacter* species (Fig. 2B) in the consortium. These data suggest that the *Trichodesmium* consortium originally isolated from the North Atlantic Ocean formed a complex symbiotic microorganism community together with the dominant *Trichodesmium* species in in Fe-depleted YBC-II medium under the conditions in our laboratory.

### Siderophores secreted by *Synechococcus* induced more severe Fe deficiency of *Trichodesmium*

Interestingly, *Synechococcus* sp. PCC 7002, a siderophore-producing strain, was found to be a symbiont of *Trichodesmium*. *Synechococcus* sp. PCC 7002 was previously believed to mainly inhabit offshore regions, but recent analysis revealed that it is also distributed in the open ocean [56, 63]. When facing Fe limitation, *Synechococcus* sp. PCC 7002 can biosynthesize an amphiphilic siderophore, synechobactin, to chelate Fe, which aids in adaptation to Fe deficiency [64]. The siderophores can be secreted to the extracellular filtrate through the type I secretion system HlyB-HlyD-TolC, and the siderophore-chelated Fe can be transported into cells through TonB-ExbB-ExbD transporter components and utilized by *Synechococcus* sp. PCC 7002 in Fe-deficient environments. However, the effect of *Synechococcus* sp. PCC 7002 siderophore secretion on *Trichodesmium* and its consortium is not clear. Some genes encoding TonB-ExbB-ExbD transporter components are present in *Trichodesmium* genomes, while TBDT, the outer membrane TonB-dependent transporter, is absent [50, 51].

**Figure 3:**
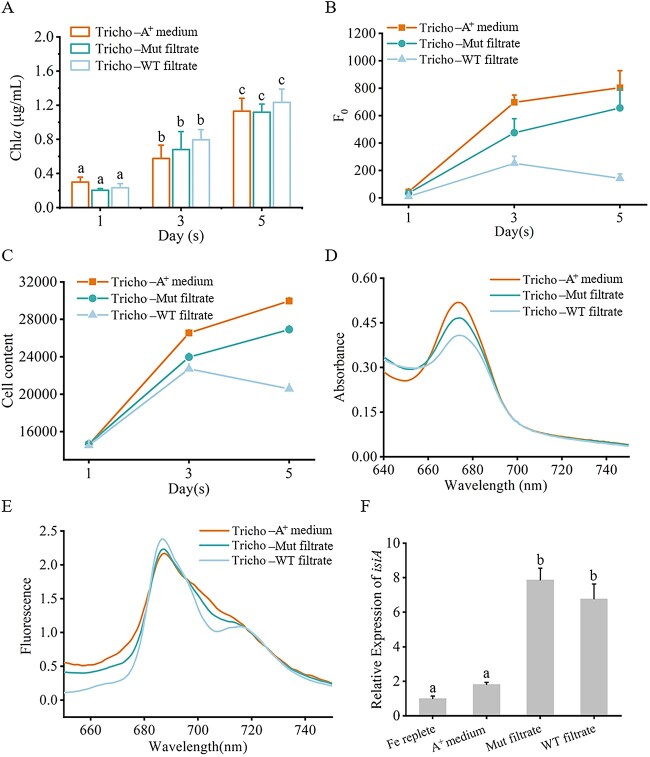
*Trichodesmium* physiological differences: Treated with *Synechococcus* secretion versus A^+^ medium. (A) Chlorophyll *a* content (μg/mL), (B) chlorophyll *a* fluorescence, (C) cell content, (D) absorption spectra at room temperature, (E) 77 K low-temperature fluorescence emission spectroscopy (excitation wavelength at 430 nm), (F) *Trichodesmium isiA* expression among three treatments. Significance difference analysis was performed using the *t* test. same and different letter labeling represents no significant difference and significant difference, respectively (Tukey’s HSD, *p* < 0.05, n = 3). Tricho-A^+^medium, Tricho-Mut filtrate, and Tricho-WT filtrate represent *Trichodesmium* in Fe deficiency with added a ^+^ medium, mutant *Synechococcus* filtrate, and wild-type *Synechococcus* filtrate, respectively.

To detect whether siderophores secreted by *Synechococcus* can be utilized by *Trichodesmium* to assist in Fe-deficient environmental adaptation, a *Synechococcus* strain knocking out mutant *G0023-G0025* (Mut-G0023-G0025) was constructed (Figure S2). We explored the interactions between wild-type or mutant *Synechococcus* strains and *Trichodesmium* in Fe-deficient environments. The Mut-G0023–25 knockout mutant (unable to biosynthesize siderophores) and wild-type *Synechococcus* sp. PCC 7002 were cultured under Fe-depleted conditions (5 nmol/L Fe) for 1 week. The *Synechococcus* culture systems were then filtered through a 0.22-μm filter membrane twice to collect filtrate (exudates of *Synechococcus* sp. PCC 7002), followed by the addition of this filtrate into *Trichodesmium* grown in Fe-depleted medium. Meanwhile, a control was set by adding the same solution of Fe-depleted A^+^ medium (5 nmol/L Fe) into Fe-depleted *Trichodesmium* culture. Results showed no significant difference in *Trichodesmium* chlorophyll *a* content among the three different treatments (Fig. 3A). Other physiological parameters, including cell contents, chlorophyll *a* fluorescence, and *Trichodesmium isiA* expression showed that the *Synechococcus* filtrate from either wild-type or mutant strains resulted in even more severe Fe-deficiency of *Trichodesmium*. Comparing the effects of wild-type filtrate and mutant filtrate on *Trichodesmium* culture, it can be found that the addition of wild-type *Synechococcus* sp. PCC 7002 filtrate resulted in a more severe Fe-deficiency to *Trichodesmium*, as shown in the cell counts. This finding suggested that the siderophores secreted by *Synechococcus* cannot aid *Trichodesmium* to adapt to Fe deficiency, and the Fe chelated by siderophores secreted by *Synechococcus* is likely not to be used by *Trichodesmium* (Fig. 3). Given that the *Trichodesmium* culture includes multiple symbionts rather than a single species of pure culture, we speculate that the siderophores produced by *Synechococcus* may exert an overall inhibitory influence on the *Trichodesmium* consortium.

### Addition of *Synechococcus* filtrate results in a decrease of relative *Trichodesmium* abundance

**Figure 4:**
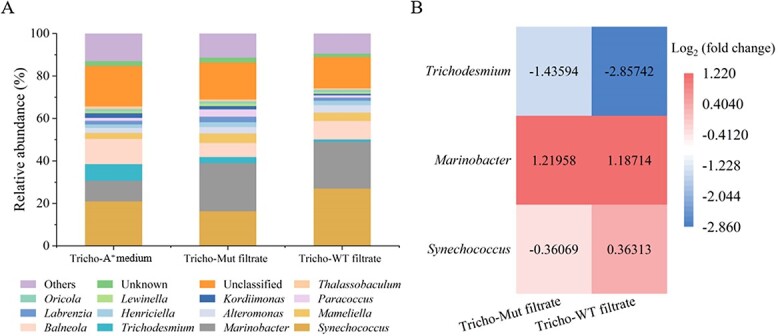
Differences in *Trichodesmium* colony community composition between treatments. (A) Relative abundance of microorganism in *Trichodesmium* colonies. “Others” represent microorganisms with relative abundance levels below 1%. (B) Difference between the addition of wild-type *Synechococcus* (with siderophores) and mutant filtrate (without siderophores) in the abundance of dominant microorganisms compared with the control, that is, the treatment with added a ^+^ medium.

To determine whether the *Synechococcus* filtrate inhibited the *Trichodesmium* consortium as a whole or merely inhibited *Trichodesmium*, metagenomic sequencing was conducted after Fe-depleted *Trichodesmium* was treated with A^+^ medium and *Synechococcus* filtrate for 5 days. Generally, cyanobacteria secrete numerous extracellular substrates, including proteins, lipids, short-chain fatty acids, and others via efflux systems [65–68]. We speculated that the *Synechococcus* exudates at late logarithmic growth (cultured under Fe-depleted conditions for 1 week) were of higher concentrations, including organic matter in addition to inorganic ions, than exudates at the beginning of the exponential phase of growth. Addition of the filtrate induced significant changes in the relative abundance of dominant organisms compared with the control group (Fig. 4). For example, the relative abundance of *Trichodesmium* decreased, while the abundance of one of the main symbionts, *Marinobacter*, increased (Fig. 4A and B). Compared to the blank control treated with A^+^ medium, the filtrate secreted by both wild-type and the mutant *Synechococcus* contained more organic matter, which might cause a great advantage over heterotrophic microorganisms such as *Marinobacter* rather than photoautotrophic *Trichodesmium* (Fig. 4A and B). The increase in the relative abundance of *Marinobacter* inevitably led to a decrease in the abundance of other related organisms within the community, including *Trichodesmium*. In addition, the change in the abundance of *Trichodesmium* induced by wild-type filtrate was more significant than the change induced by the mutant filtrate (Fig. 4B). Siderophores contained in wild-type filtrate are likely beneficial for the growth of symbionts which complete with siderophore transporter components [69] such as *Marinobacter*. Furthermore, siderophores contained in the filtrate of wild-type *Synechococcus* may competitively adsorb the free inorganic Fe required for *Trichodesmium* in the medium and result in more severe *Trichodesmium* Fe deficiency. *Trichodesmium* consortia and *Synechococcus* filtrate contents and inhibitory mechanisms need to be further explored to explain these phenomena.

### Global biological community processes are promoted by *Synechococcus* filtrate

**Figure 5:**
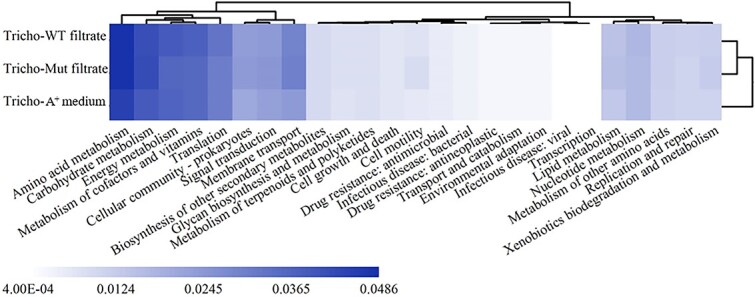
Changes in the relative abundance of genetic functions among different treatments as shown with a heatmap. The top 25 functional categories in the sample in terms of average abundance are shown, with abundance characterized by color depth, the darker the color, the larger the value.

We explored the changes in functional gene abundance by analyzing metagenomic sequencing data to ascertain the reasons for the increased severity of *Trichodesmium* Fe deficiency after adding wild-type *Synechococcus* filtrate. As shown in the heatmap (Fig. 5), almost all functional categories within the consortium increased in the samples treated with *Synechococcus* filtrate regardless of the presence of siderophores compared with the samples treated with A^+^ medium. These data suggest that complex substrates contained in *Synechococcus* filtrate may be the major reason for the promotion of global biological community processes (Fig. 5), which is consistent with the observed increase in *Synechococcus* and *Marinobacter* relative abundances (Fig. 4A). Additionally, filtrate promotion was more pronounced for some basal consortium metabolisms, such as amino acid metabolism, carbohydrate metabolism, membrane transport, and replication and repair (Fig. 5); however, these results cannot explain why *Trichodesmium* Fe deficiency is more severe, as the data from metagenomic sequencing cannot reflect which organisms encode for those functional genes whose abundances have changed.

### *Trichodesmium* metabolism is inhibited whereas *Marinobacter* metabolism is promoted by *Synechococcus* filtrate

**Figure 6:**
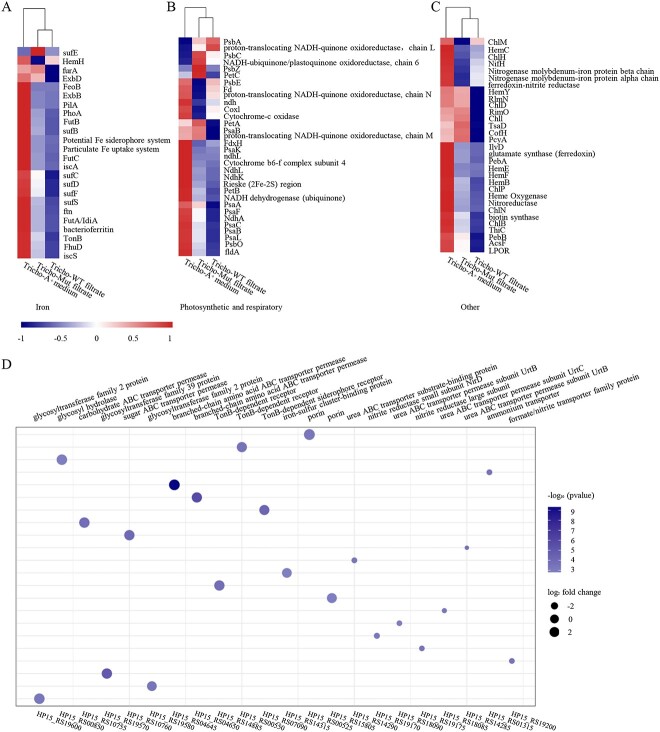
Expression changes of *Trichodesmium*-related and *Marinobacter*-related genes. Heatmap of the expression abundance of genes involved in Fe absorption and utilization (A), photosynthesis and respiration (B), and other essential metabolic pathways (C) in *Trichodesmium* treated by A^+^medium, *Synechococcus* wild-type filtrate, and mutant filtrate. Expression abundance of genes is indicated by color depth. (D) Changes in expression abundance of related genes of *Marinobacter* under treated with filtrate from wild type compared to the A^+^ medium treatment. The size and the color of the dot represents the fold change and *p* value, respectively.

To further explore changes in the expression abundance of functional *Trichodesmium* genes, we conducted metatranscriptome sequencing after treatment with *Synechococcus* filtrate or A^+^ medium for 5 days. The *Trichodesmium* erythraeum IMS 101 genome was used as a reference genome to analyze the sequencing data. By analyzing the expression abundance of genes that relate to Fe absorption and transport, photosynthesis and respiration, and other essential metabolic pathways of *Trichodesmium*, we found that for most of these genes the expression abundances decreased after *Synechococcus* filtrate treatment compared with treatment with A^+^ medium, demonstrating that the substrates in filtrate inhibited *Trichodesmium* (Fig. 6A, B and C). Genes related to *Trichodesmium* Fe uptake, transport, and storage were downregulated after treatment with wild-type and mutant strain filtrates (Fig. 6A). Comparing the effects of the filtrates from the wild-type and mutant strains, we found that the expression of the genes related to Fe-deficiency adaptation was much lower in the wild-type–treated group, including *tonB*, *exbB*, *exbD*, *sufB*, *sufC*, *sufD*, *sufF*, *futA*, and *futB* (Fig. 6A). Furthermore, genes related to *Trichodesmium* photosynthesis and respiration (such as *psaA*, *psaF*, and NADH–ubiquinone/plastoquinone oxidoreductase genes) and genes related to various essential metabolic pathways (including nitrogen fixation, RNA modification, and amino acid synthesis) were also downregulated after *Synechococcus* secretion filtrate treatment (Fig. 6B and C), suggesting the filtrate affects *Trichodesmium* photosynthesis, respiration, and global metabolism, consistent with observed physiological phenotype.

Because *Marinobacter* abundance increased in the sample treated with *Synechococcus* filtrate, the Marinobacter adhaerens genome was used as a reference genome, as was the *Trichodesmium* erythraeum IMS 101 genome, in our metatranscriptome analysis. Results showed that *Marinobacter* genes related to nitrogen utilization (e.g., urease, nitrite reductase, and urea transport) were significantly downregulated in the sample when *Synechococcus* filtrate was added (Fig. 6D). This finding illustrated a reduction of directly available nitrogen sources in the environment and was consistent with decreased *Trichodesmium* abundance. In contrast, genes relevant to *Marinobacter* TonB–dependent siderophore receptors, sugar utilization, and amino acid utilization are significantly upregulated (Fig. 6D), indicating that the rich organic matter and siderophore chelated Fe in the filtrate are beneficial for *Marinobacter* growth and lead to an increase in its relative abundance. *Marinobacter* consumed Fe and further reduced the concentration of Fe. In addition, siderophores secreted by the *Synechococcus* wild-type strain are likely to chelate with free Fe in the medium of the *Trichodesmium* consortium, reducing the concentration of bioavailable Fe and posing a greater challenge for *Trichodesmium* to obtain Fe.

## Discussion

Recent research revealed that the habitats of major nitrogen-fixing and siderophore-producing cyanobacteria are gradually overlapping [56]. In addition, the presence of closely related *Synechococcus* species in environmental *Trichodesmium* samples from both the Sargasso Sea and the southwest Pacific Ocean indicated that *Synechococcus* can be symbiotic with *Trichodesmium*, which as a nitrogen-fixing cyanobacterium and is known for its widespread occurrence in vast oligotrophic subtropical and tropical oceans in these habitats [11, 70–72]. In particular, our recent analysis of *Tara* ocean data shows the widespread distribution of siderophore-producing *Synechococcus* species both offshore and in pelagic ocean (which were previously thought to be mainly distributed offshore) [56, 73]. In Fe-deficient ocean, some *Synechococcus* such as *Synechococcus* sp. PCC 7002 can synthesize and secrete siderophores, which can bind inorganic free Fe^3+^ in the environment and then be transported back into the cells through TonB-dependent transporters (TBDTs) mediated by TonB-ExbB-ExbD components [56, 64]. The special Fe uptake mechanism provides siderophore-producing *Synechococcus* a high advantage in Fe-deficient ocean [56]. The interactions between nitrogen-fixing and siderophore-producing cyanobacteria in oceans may have profound implications for the contribution of primary productivity and elemental cycling in the oceans.

Fe deficiency is one of the limiting factors of *Trichodesmium* in oligotrophic open ocean [17]. As a diazotrophic cyanobacterium that cannot produce siderophore but has TonB-ExbB-ExbD transporter components, *Trichodesmium* may adapt to Fe deficiency by absorbing siderophore secreted by its epibionts [50]. of the study by Basu et al. [48] revealed enhanced Fe uptake by *Trichodesmium* in the presence of desferrioxamine B (DFO-B) and desferrioxamine E (DFO-E), while the Fe uptake of *Trichodesmium* was inhibited when only DFO-B was provided [48, 53]. The various utilization of different siderophores may be related to the different chemistries of siderophores as well as the utilization of different forms of Fe by symbionts. Given the specific Fe uptake mechanism, *Synechococcus* may have a higher competitive advantage in Fe-deficient pelagic ocean, and the interaction between *Synechococcus* and *Trichodesmium* may have a great impact on future marine primary productivity. To simulate the interaction between *Trichodesmium* and *Synechococcus* in Fe-deficient ocean water, *Trichodesmium* have been chronically grown in Fe-depleted medium for more than 2 months before treated with *Synechococcus* filtrate. By comparing the responses of *Trichodesmium* to the filtrates with or without synechobactin or not, we found that the growth rate of *Trichodesmium* slows down, photosynthetic efficiency is decreased, and the expression of *isiA* is increased—all demonstrating that the filtrates that contained synechobactin were likely to inhibit the growth of *Trichodesmium* under Fe-deficient conditions and induced a more severe Fe deficiency in *Trichodesmium*. Simultaneously, the relative abundance of *Trichodesmium* decreased, especially in the group treated with the wild-type *Synechococcus* filtrate that contains siderophores. The possible reason for this finding can be attributed to the observation that the siderophores produced by *Synechococcus* are absorbed by other symbionts with complete siderophore transporter components or stimulate the symbionts to produce siderophores and other products, which in turn exerted further survival pressure on *Trichodesmium* under Fe-deficient conditions. Furthermore, *Synechococcus* abundance variation is opposite that of *Trichodesmium* abundance variation, indicating a negative correlation between them in this consortium, which is similar to a recent discovery [74]. *Synechococcus* is outcompeted when diazotrophs increase the NH_4_^+^/NO_3_^−^ ratio (the main nitrogen produced by the fixation of N_2_ by *Trichodesmium* is NH_4_^+^), which favors *Prochlorococcus*, suggesting a different competitive pattern between nitrogen-fixing cyanobacterium and *Synechococcus* [74]. Interactions between cyanobacteria and associated symbionts are quite complex [75, 76], and there is a lot of work to be done to figure out the complex interactions.

**Figure 7:**
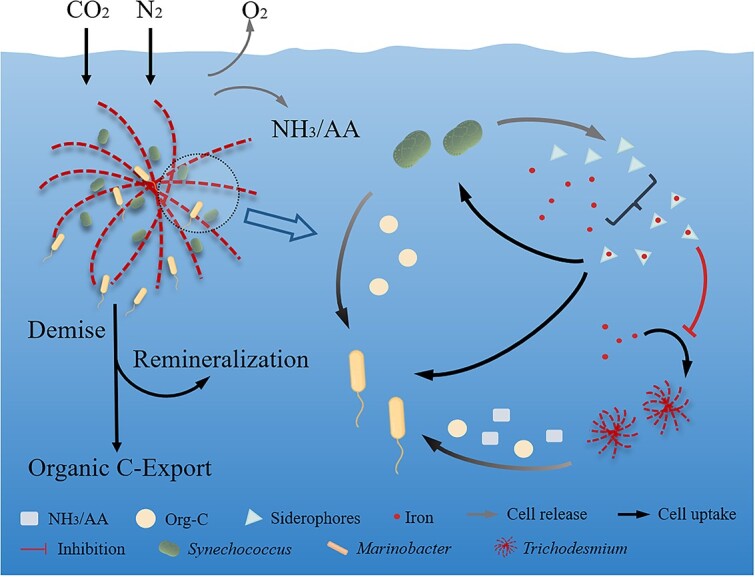
Putative interaction of *Trichodesmium* consortium under Fe deficiency. NH_3_/AA, ammonium nitrogen/amino acid; Org-C, organic carbon.

Metagenomic and metatranscriptomic analyses show that treatment with both *Synechococcus* filtrates reduces the abundance of *Trichodesmium* but promotes global *Trichodesmium* consortium metabolism, especially in samples treated with wild-type *Synechococcus* filtrate containing siderophores. This finding may be due to the filtrate being rich in siderophores and organic matter that can be utilized directly by heterotrophic *Marinobacter* [54, 77, 78]. Functional gene expression abundance analyses in our study indicate that the metabolism of the symbiotic microorganisms is enhanced, while the metabolism of *Trichodesmium*, including its Fe transport and utilization, photosynthesis, respiration, and other essential metabolic mechanisms are actually downregulated, which is consistent with the physiological phenotype seen in Fe-deficient *Trichodesmium*. These data collectively demonstrate that the siderophores produced by *Synechococcus* chelate most of the available free Fe in the medium, which is then used by the symbionts such as *Synechococcus* and heterotrophic microorganisms (Fig. 7). The cell size of *Trichodesmium* is large, thus the specific surface area is smaller than that of *Synechococcus* and heterotrophic microorganisms. The smaller specific surface area offers them quite an advantage for absorbing Fe and adapting to an environment with low Fe concentrations when the concentration of Fe in the environment decreases [57]. Once other organisms are taken up and lower the concentration of Fe, it becomes harder for *Trichodesmium* to take in Fe and makes Fe deficiency in *Trichodesmium* even worse, inhibiting the growth of *Trichodesmium* (Fig. 7). This phenomenon is consistent with previous research demonstrating that *Trichodesmium* can most readily access inorganic Fe or Fe in association with weak organic ligands but not strongly bound organic complexes under Fe-limited culture conditions [35]. In addition to siderophores, *Synechococcus* filtrate also contains various organic matters and extracellular proteins [77, 79]. These can serve as Fe, carbon, and other nutrient sources to be directly used by heterotrophic symbionts, such as *Marinobacter*, and serve as a primer to stimulate and expedite *Marinobacter* growth (Fig. 7). Within this context, increased *Marinobacter* and *Synechococcus* abundances further compete with *Trichodesmium*, which poses a disadvantage for *Trichodesmium* and deccreases its relative abundance. The production of other siderophores and products from symbionts after treating with *Synechococcus* filtrate is another possibility to explain the inhibition of *Trichodesmium* growth, while the way these substrates function in *Trichodesmium* consortium is unknown and makes the interaction more complex.

Our findings revealed the inhibition of *Synechococcus* siderophore to *Trichodesmium* using culture interaction experiments in the laboratory. This finding highlighted the observation that Fe chelated by siderophores secreted by *Synechococcus* exerts an inhibitory influence on *Trichodesmium* and defines the competitive relationship in Fe utilization between *Synechococcus* and *Trichodesmium* in Fe-deficient habitats. These microorganisms have been in culture for long time and whether these interactions will be sustained in the more complex and dynamic ocean environment needs to be investigated in the future. But at least microorganisms such as *Synechococcus* and *Marinobacter* can be found in many field metagenome data [18]. Since interactions between different species in the consortium are quite complex, more field work is needed to elucidate the interaction between *Trichodesmium* and symbionts in nutrient acquisition and environmental adaptation.

## Supplementary Material

FigureS1_ycae072

FigureS2_ycae072

Supplementary_figures_ycae072

## Data Availability

The datasets generated during the current study are available in the NCBI repository under accession number PRJNA903535 (ID 903535 - BioProject - NCBI (nih.gov)).
