# Supplementary figures and images for: Interactions between the nitrogen-fixing cyanobacterium Trichodesmium and siderophore-producing cyanobacterium Synechococcus under iron limitation
Source: ISME Commun. 2024 May 25;4(1):ycae072. doi: 10.1093/ismeco/ycae072 (PMC11171426; doi:10.1093/ismeco/ycae072)

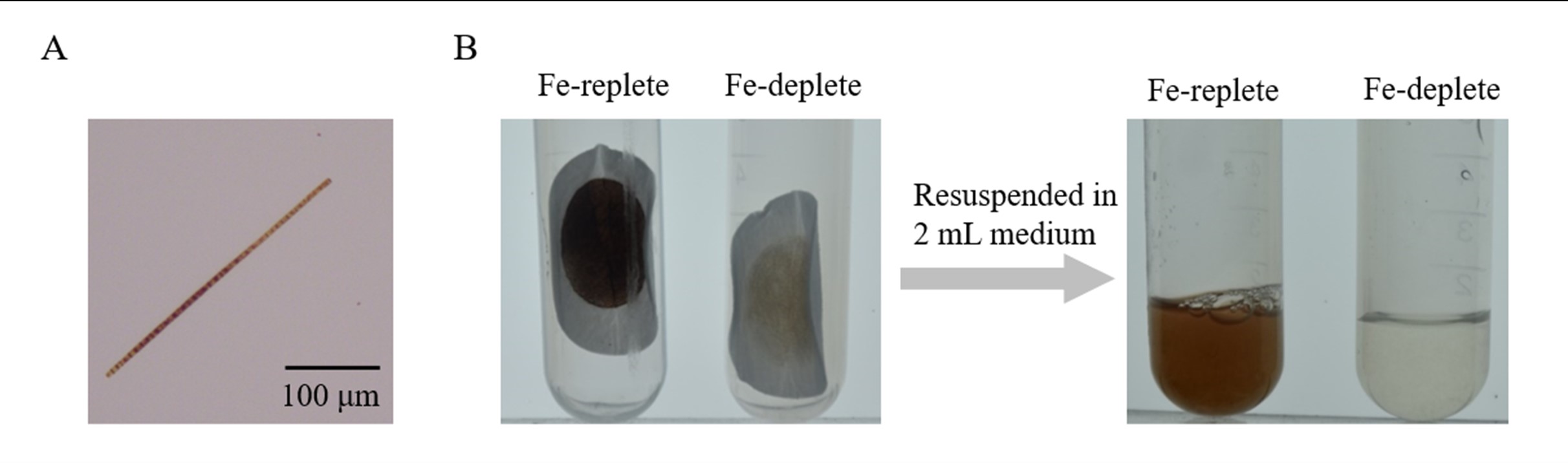

Supplement: FigureS1_ycae072 [file figures1_ycae072.jpeg]

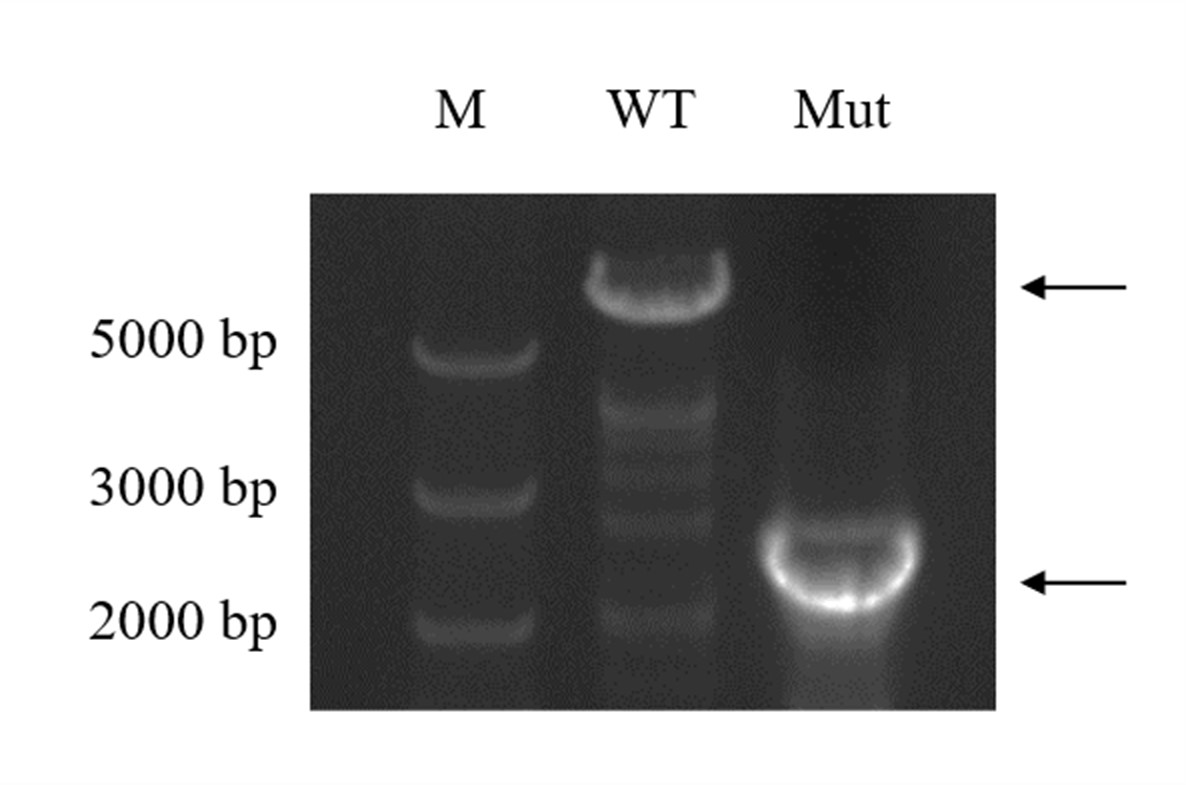

Supplement: FigureS2_ycae072 [file figures2_ycae072.jpeg]
