# Supplementary material for: Interactions between the nitrogen-fixing cyanobacterium Trichodesmium and siderophore-producing cyanobacterium Synechococcus under iron limitation
Source: ISME Commun. 2024 May 25;4(1):ycae072. doi: 10.1093/ismeco/ycae072 (PMC11171426; doi:10.1093/ismeco/ycae072)
Supplement: Supplementary_figures_ycae072 [file supplementary_figures_ycae072.doc]

**Supplementary figures**

**Figure S1. *Trichodesmium* under Fe-deficient cultivation.** (A) *Trichodesmium* light microscopy photograph, scale bar: 100 µm. (B) *Trichodesmium* biomass after 10 days of cultivation. 100 mL of liquid algal culture was collected by filtering and resuspended in 2 mL YBC-II medium.

**Figure S2. *Synechococcus* sp. PCC 7002 Mut-G0023-25 gene cluster knockout strain detection. “**M,” “WT,” and “Mut” represent marker, wild-type *Synechococcus* sp. PCC 7002, and the *Synechococcus* sp. PCC 7002 knockout strain, respectively. Arrows indicate the target gene fragment.
